# A Primary Survey on Bryophyte Species Reveals Two Novel Classes of Nucleotide-Binding Site (NBS) Genes

**DOI:** 10.1371/journal.pone.0036700

**Published:** 2012-05-15

**Authors:** Jia-Yu Xue, Yue Wang, Ping Wu, Qiang Wang, Le-Tian Yang, Xiao-Han Pan, Bin Wang, Jian-Qun Chen

**Affiliations:** State Key Laboratory of Pharmaceutical Biotechnology, School of Life Sciences, Nanjing University, Nanjing, Jiangsu Province, China; University of Illinois, United States of America

## Abstract

Due to their potential roles in pathogen defense, genes encoding nucleotide-binding site (NBS) domain have been particularly surveyed in many angiosperm genomes. Two typical classes were found: one is the TIR-NBS-LRR (TNL) class and the other is the CC-NBS-LRR (CNL) class. It is seldom known, however, what kind of NBS-encoding genes are mainly present in other plant groups, especially the most ancient groups of land plants, that is, bryophytes. To fill this gap of knowledge, in this study, we mainly focused on two bryophyte species: the moss *Physcomitrella patens* and the liverwort *Marchantia polymorpha*, to survey their NBS-encoding genes. Surprisingly, two novel classes of NBS-encoding genes were discovered. The first novel class is identified from the *P. patens* genome and a typical member of this class has a protein kinase (PK) domain at the N-terminus and a LRR domain at the C-terminus, forming a complete structure of PK-NBS-LRR (PNL), reminiscent of TNL and CNL classes in angiosperms. The second class is found from the liverwort genome and a typical member of this class possesses an α/β-hydrolase domain at the N-terminus and also a LRR domain at the C-terminus (Hydrolase-NBS-LRR, HNL). Analysis on intron positions and phases also confirmed the novelty of HNL and PNL classes, as reflected by their specific intron locations or phase characteristics. Phylogenetic analysis covering all four classes of NBS-encoding genes revealed a closer relationship among the HNL, PNL and TNL classes, suggesting the CNL class having a more divergent status from the others. The presence of specific introns highlights the chimerical structures of HNL, PNL and TNL genes, and implies their possible origin via exon-shuffling during the quick lineage separation processes of early land plants.

## Introduction

Plant disease resistance (*R*) genes are a set of genes that confer resistance to various invading pathogens. For example, the tobacco *N* gene can prevent invasion by the *Tobacco Mosaic Virus* [1], [2], [3], [4], the *RPW8* gene in *Arabidopsis thaliana* provides resistance to mildew caused by *Erysiphe cruciferarum* infection [5] and the *Pik* gene confers resistance to rice blast, which is caused by *Magnaporthe grisea* [6]. Owing to the potential connection between disease resistance and economic crop production, many efforts have been devoted to the functional study of *R* genes. Meanwhile, investigating the origin and evolution of *R* genes has attracted the attention of evolutionists [7], [8], [9], [10], [11], [12]. Among all types of *R* genes in plants, genes encoding the nucleotide-binding site (NBS) domain form the largest group. Genome-wide analyses have been performed on many angiosperms, including *Arabidopsis thaliana, Brachypodium distachyon, Brassica rapa, Glycine max, Medicago truncatula, Oryza sativa, Populus trichocarpa, Sorghum bicolor, Vitis vinifera* and *Zea mays* [13], [14], [15], [16], [17], [18], [19]. All these surveyed genomes were found to contain a large number of NBS-encoding genes, offering invaluable information on the evolutionary dynamics of these disease-defending genes in angiosperms.

With a chimerical structure, a typical NBS-encoding gene consists of an amino (N)-terminal domain, an NBS domain in the middle and a leucine-rich repeat (LRR) domain near the carboxy(C)-terminus. However, truncated NBS-encoding genes lacking LRR domain are not rare. It has been estimated that less than 80% of NBS-encoding genes in angiosperm genomes exist in intact NBS-LRR form [13], [14], [15], [17], [18], [19]. Within the NBS domain, a number of small motifs with 10–30 amino acids in length are recognized [20]. From the N-terminus to C-terminus, they appear in the order of P-loops, RNBS-A, Kinase-2, RNBS-B, RNBS-C, GLPL, RNBS-D and MHDV [19]. Functionally, the NBS domain converts ADPs into ATPs after the recognition of alien pathogen signals, and thus activates the downstream hypersensitive resistance reaction [21], [22], [23], [24], [25]. Based on the identity of N-terminal domain, NBS-LRR genes can be further divided into different classes: a typical one is the TIR-NBS-LRR (TNL) class, which has an N-terminal domain sharing high similarity to known Toll/Interleukin-1 Receptor protein. The rest are grouped into nonTIR-NBS-LRR (nonTNL) class, with most members owning a coiled-coil domain (CC). For this reason, nonTNL class are often regarded as CC-NBS-LRR (or CNL) class, although in a strict way, CNL only represents a part of nonTNL class in angiosperms.

Almost all current knowledge on NBS-encoding genes came from investigations on angiosperm genomes, with very little attention paid to other clades of land plants. In particular, the lack of knowledge regarding NBS-encoding genes in early-diverging lineages of land plants, such as bryophytes, has to a large extent hampered our understanding of the origin and evolutionary history of this important type of *R* genes. Our previous investigations into the genomes of bacteria, archaea, protists, and algae have shown that the NBS domain had not combined with the LRR domain yet [26]. The origin of NBS-LRR genes are therefore hypothesized to coincide with the process of plants colonizing the land.

To test the hypothesis, one good entry point can be the recently published genome of the moss *Physcomitrella patens* [27]. In land plant phylogeny, the mosses emerged far earlier than angiosperms (about 450 million years ago for mosses and 100 million years ago for angiosperms). Thus the *P. patens* genome offered us a great opportunity to investigate whether mosses have harbored an ancestral status of NBS-LRR genes and to see what these genes look like. Besides the moss *P. patens*, data from the earliest-diverging lineage of land plants liverworts [28], [29], would further help to elucidate the issue. Therefore, we also focused on a liverwort, *Marchantia polymorpha*, to experimentally isolate NBS-encoding genes from this complex thalloid liverwort. Surprisingly, the obtained results demonstrated that a diversity of NBS-encoding genes existed in early land plants, including two novel classes.

## Results

### Surveying of the NBS-encoding Genes in the Moss *P. Patens* Genome

Through BLAST and HMM (Hidden Markov Model) searches, a total number of 65 NBS-encoding genes were identified from the *P. patens* genome. These NBS-encoding genes, however, are not unanimous in structure (Table 1). Only 18 of them are intact, with N-terminal domain, NBS domain, and LRR domain all present, while the remaining 47 NBS-encoding genes either lack an N-terminal domain (7), a LRR domain (20), or lack both (20).

**Table 1:** Number of NBS-encoding genes identified from the *Physcomitrella patens* genome and their domain compositions.

Class	Domain	Number	Total
TNL	TNL	3	9
	TN	0	
	NL	5	
	N	1	
PNL	PNL	6	45
	PN	18	
	NL	2	
	N	19	
CNL	CNL	9	11
	CN	2	
	NL	0	
	N	0	

Among the 18 intact ones, three belong to the TNLs; nine belong to the CNLs; and the remaining six, each possess a protein kinase (PK) domain at the N-terminus. By using a similar naming system for TNL and CNL classes, for convenience, we call the new type of PK-NBS-LRR genes as PNL class. Among the 47 shortened NBS-encoding genes, 39 were found to share high sequence similarities to the six PNL genes at NBS domain, making the total member of this new class 45, about two-thirds of all the identified NBS-encoding genes in this moss genome. In addition, six shortened NBS-encoding genes were classified with TNLs and two were with CNLs (Table 1).

### Isolation of NBS-encoding Genes from the Liverwort *M. Polymorpha*

**Figure 1:**
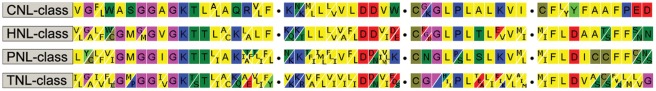
Conserved motifs of the NBS domains of the four classes of NBS-encoding genes. The four motifs are separated by dots and are in the following order: P-loop, kinase 2, GLPL and RNBS-D.

To investigate whether liverwort (another bryophyte lineage diverged earlier than mosses in land plant phylogeny, [29]) also had a large number of PNLs in their genomes, we focused on a common liverwort species *M. polymorpha* to amplify its NBS-encoding gene fragments using a combination of primers (Table S1). Gel-purified PCR products were further cloned. Collectively, 416 clones were picked up and sequenced. 389 obtained sequences are homologous to NBS domain. After assembly and editing, a total of 43 non-redundant NBS-encoding genes were identified. 42 of them have normal reading frames while only one possesses an internal stop codon, indicating a pseudolization event. Seven of the obtained NBS sequences share high sequence similarity with the *P. patens* CNLs; however, the remaining 36 genes did not seem to belong to any of the three known classes (TNL, CNL, or PNL). Careful comparison of the small motifs within the NBS domain found that the RNBS-A, RNBS-B and RNBS-C motifs of the 36 genes show lower sequence similarity to the corresponding motifs of the TNL, CNL and PNL classes than the other motifs like P-loop, Kinase 2, GLPL and RNBS-D, which seem more conserved among all classes (Figure 1).

**Figure 2:**
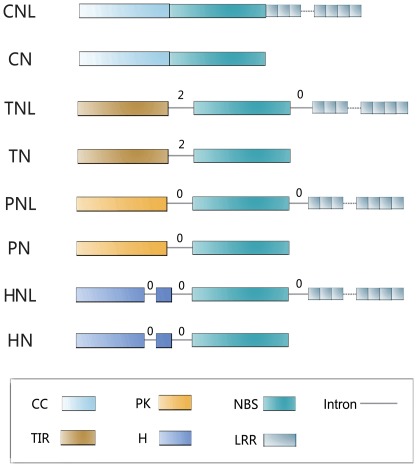
The intron positions and phases of the four classes of land plant NBS-encoding genes.

### RACE Helped to Identify the N-terminal Domains and the LRR Domain of *M. Polymorpha* NBS-encoding Genes

To aid in the classification of the 36 unique NBS homologs in *M. polymorpha*, we carried out rapid amplification of 5′ cDNA ends (5′-RACE) to identify their N-terminal domains and successfully obtained the N-terminal domain sequences for nine selected NBS-encoding genes. The obtained N-terminal sequences share moderate (>40% at amino acid level) to high (>80% at amino acid level) similarities with each other and are highly conserved at specific regions, which might be important functional motifs. Pfam analyses attribute these N-terminal regions as homologs of α/β-hydrolase domain. Meanwhile, the 3′-RACE identified C-terminal LRRs from three of the nine selected NBS-encoding genes. Together these findings suggested that the nine genes tested all belong to a same, previously unknown class of NBS-encoding genes, with at least three members maintaining complete structures with N-terminal domain, NBS domain, and LRR domain all present. To be distinguished from the TNL, CNL and PNL classes, we named this new type of NBS-encoding genes in the liverwort *M. polymorpha* as α/β-hydrolase-NBS-LRR (HNL) class. All the 36 NBS-encoding genes can be reasonably grouped into this HNL class due to their highly similar sequences at NBS domain, although some members may be actually truncated NBS-encoding genes lacking either the LRR domain or the α/β-hydrolase domain, or even both.

3′-RACE experiments were also carried out for the seven NBS fragments homologous to CNLs. Full length sequence of C-terminus was obtained for one gene and the LRR domain was also characterized. This result proved the actual existence of CNL-like NBS-LRR gene in *M. polymorpha*.

### Analyzing the Intron Positions and Phases of NBS-encoding Genes can Help to Distinguish their Belonged Classes

To further confirm that the PNLs and the HNLs are indeed two novel classes of NBS-encoding genes different from the TNLs and the CNLs, we tried to investigate their intron characteristics. Due to a narrow coverage for only NBS domain, most of the isolated *M. polymorpha* NBS sequences contain no introns. However, a few NBS-encoding genes with longer sequences obtained via RACE do cover the N-terminal hydrolase domain region and even the C-terminal LRR region. Thus their coding sequence information could be used to design specific primers and to amplify their genomic sequences containing introns.

Interestingly, the intron positions and phases were found to be distinctive among different classes of NBS-encoding genes. The term ‘intron phase’ refers to the position of an intron within a codon: phase 0, 1 or 2 corresponds to an intron lying before the first base, after the first base and after the second base of a codon, respectively. As shown in Figure 2, TNL/TN genes have a conserved intron position between the TIR domain and NBS domain, and the phase of this intron is 2. PNL/PN class (named in this study, see above) also have a conserved intron location separating the PK domain and the NBS domain, but its phase is 0. CNL/CN genes do not have conserved intron between CC domain and NBS domain, whereas the HNL class (also named in this study) contains introns at three conserved locations: two lie before the NBS domain and one lies behind it. Of the two introns located before the NBS domain, one is between the α/β-hydrolase domain and the NBS domain and the other is within the α/β-hydrolase domain, separating it into two exons. Both the positions and phases (0) of the two introns are conserved within HNL members. In addition, we further surveyed more angiosperm NBS-encoding genes, mainly those TNL and CNL members. The results are consistent with observations in bryophytes described above. Intron positions and phases could be therefore taken as distinguishing characteristics to classify NBS-encoding genes.

### Phylogenetic Analysis of *P. Patens* and *M. Polymorpha* NBS-encoding Genes

**Figure 3:**
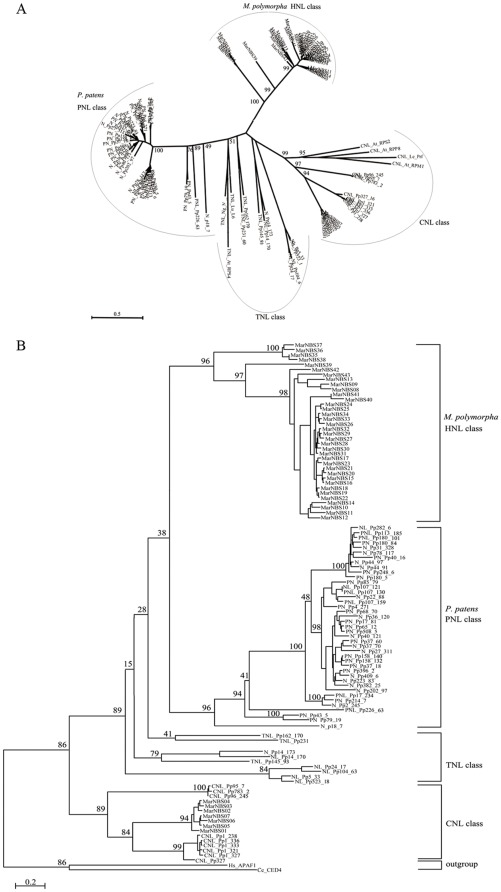
A. Unrooted phylogenetic tree of the NBS domain sequences of *Physcomitrella patens*, *Marchantia polymorpha* and seven functional angiosperm NBS-encoding genes. B. Phylogenetic tree of the NBS domain sequences of *Physcomitrella patens* and *Marchantia polymorpha* NBS-encoding genes. *Lu, Linum usitatissimum; At, Arabidopsis thaliana; Le, Lycopersicon esculentum; Hs, Homo sapiens; Ce, Caenorhabditis elegans.* The abbreviated species names are followed by the names of functional genes.

In order to understand the evolutionary relationships between and within each class of NBS-encoding genes, phylogenetic trees were constructed using the NBS domain regions of 59 *P. patens* and 43 *M. polymorpha* NBS-encoding genes. Six *P. patens* NBS-encoding genes were excluded from the matrix because they were either too short or too divergent in a fine alignment. Additionally, an unrooted phylogenetic tree was built with 7 more angiosperm NBS-LRR genes of known function included. Collectively, the 102 bryophyte NBS sequences were found to form four clusters (Figure 3A & B). Among the major clusters, all the CNL sequences from liverwort and moss species form a monophyletic group with strong support (bootstrap value 89, Figure 3B). The 4 angiosperm CNLs also fall within the monophyletic group with a high bootstrap value of 99 (Figure 3A). Within this group, the *P. patens* CNL genes could be further classified into two subgroups, and all seven obtained *M. polymorpha* CNL genes were closer to one CNL subgroup of *P. patens* than the other. Besides of CNLs, the remaining HNL, PNL and TNL genes also formed a monophyletic group sister to the CNL genes with a good bootstrap value (89, Figure 3B). Within this large group, both the PNL class from the moss *P. patens* and the HNL class from the liverwort *M. polymorpha* form monophyletic group on their own, suggesting independent expanding processes. Differently, the moss TNL genes seem to be paraphyletic (Figure 3A & B), and the 3 angiosperm TNLs locate within the T/P/HNL superclass and near all other TNLs (Figure 3A).

## Discussion

### When did the NBS Domain and LRR Domain Fuse Together?

Almost all our current knowledge on NBS-LRR genes came from studies on angiosperm species, leaving questions about the status and evolutionary dynamics of these genes in other major groups of land plants largely unexplored. For example, a basic but important question is when the NBS domain and LRR domain became fused together. Our efforts in searching genomes of bacteria, archaea, protists, and algae only detected independent NBS or LRR domains [26]. Such fact prompted us to speculate that the origin of NBS-LRR genes may accompany the period of land plant evolution. However, the direct evidence supporting this idea is lacking.

From the moss *P. patens*, Akita and Valkonen had isolated 3 NBS-encoding genes before and found that they are all homologous to the TNL class members in angiosperms [30]. This result lends support to the idea that the NBS-LRR genes have already appeared in plant genome back to the diverging time of the moss lineage (450 million years ago). Further, as liverwort lineage diverged >30 million years earlier than mosses, it is critical to know whether NBS-LRR gene had already shown up and been maintained in the liverworts.

To investigate these specific questions, in this study, we focused on two bryophyte species (one the liverwort *M. polymorpha* and the other the moss *P. patens*) to survey their genomes for possibly harbored NBS-LRR genes. As showed in the results, a total of 25 genes covering both NBS and LRR domains were identified from the *P. patens* genome (Table 1) and at least four NBS-LRR genes were amplified from the liverwort *M. polymorpha*. These data provide clear evidence to support the idea that the NBS and LRR domains have already fused together in the two earliest diverging lineages of land plants. In future, it will be interesting to explore Charophyta species to see whether NBS-LRR gene had originated even before the colonization of land by plants.

### Two Novel Classes of NBS-encoding Genes in Bryophytes: PNL and HNL

Previous studies on angiosperms have identified two major classes of NBS-encoding genes: the TNL class and the CNL class. The finding of 3 TNL genes in the moss *P. patens* [30] had suggested that at least the TNL class is very ancient. It is unknown, however, whether more NBS-LRR genes are harbored in the moss *P. patens* genome and what classes they belong to. Here, our investigations into the two bryophyte species not only revealed that the CNL class is also ancient, with members presented in both the *P. patens* and *M. polymorpha*, but also unexpectedly discovered two novel classes of NBS-LRR genes, as represented by six intact PNL genes in *P. patens* genome and at least three intact HNL genes amplified from *M. polymorpha*. ESTs have been found for both PNLs (FC330793.1, FC356926.1, FC370257.1) in *P. patens* and HNLs (BJ861543.1, BJ865527.1, BJ861316.1) in *M. polymorpha* from GenBank, and our RACE results also proved that some HNLs in *M. polymorpha* were transcribed, which means these novel genes are not errors of genomes assembly or non-functional pseudogenes.

For those identified NBS-encoding genes lacking a complete structure, classification mainly relied on the sequence similarity tests and phylogenetic analysis using NBS domain (see next section). Previously, Meyers and his colleagues had found that the sequences of NBS domain can be effectively used to distinguish the TNL and the CNL classes in angiosperms [8]. Here we found that this strategy can also be applied on the PNL and the HNL classes in bryophytes, with 39 more PNL-like shortened sequences identified in *P. patens* and 36 HNL-like sequences in *M. polymorpha*. Thus, the PNL class becomes the most dominant class in *P. patens* (45/65) and the HNL the major class in *M. polymorpha* (36/43).

Such dominant status seems also hold true in other liverwort and moss species (unpublished data). Primary data obtained from other bryophyte species, including *Treubia lacunose* and *Polytrichum juniperinum* (the very basal taxa of liverworts and mosses, respectively), consistently support the dominance of the HNL class in liverworts and the PNL class in mosses. We neither amplified any PNL members from liverwort species nor obtained any HNL-like sequences from moss species. Moreover, neither the PK domain nor the α/β-hydrolase domain has ever been discovered together with an NBS domain in angiosperm genomes. This suggests that the HNL is a liverwort lineage-specific class and the PNL is a moss-lineage specific class. Future studies covering a diverse group of taxa could supply more convincing results on this issue.

So far, little is known about why and how the PK domain and the hydrolase domain became fused with NBS-LRR genes in bryophytes. PK domain is found in various other proteins, including receptor-like kinases and receptor-like proteins, both of which contain LRRs. The α/β-hydrolase domain also belongs to a super-family, which consists of dozens of proteins sharing lower sequence conservation. It is logical to speculate that during the early evolution of bryophytes, the PK domain and the hydrolase domain, likely via exon-shuffling events, became associated with NBS-LRR gene respectively in liverwort and moss lineages. The fused genes could then replicate, experience structural changes, and have at least some members armed with new functions, otherwise they would not have been retained over such a long evolutionary period.

### Intron Characteristics and Phylogenetic Analyses Revealed a Closer Relationship among HNL, PNL and TNL Classes

Intron characteristics have been recognized as an important aid in resolving difficult relationships of land plant lineages [28], [31], [32]. The present/absent status, position and phase of introns can provide useful, sometimes critical, information on gene evolution as well. On the case of NBS-LRR genes, Meyers and his colleagues had discussed the intron characteristics when surveying the *Arabidopsis thaliana* genome [19]. Since then, no further intron analyses were conducted although many other plant genomes had also been surveyed for their NBS-LRR genes. Here in this study, we performed the intron position and phase analyses on obtained NBS-encoding genes from *P. patens* and *M. polymorpha* genomes, as well as on NBS-encoding genes from five angiosperm genomes (*A. thaliana, M. truncatula, O. sativa, P. trichocarpa,* and *S. bicolor*). Our results showed that NBS-encoding genes of HNL, PNL, and TNL classes all maintain their class-specific intron characteristics. As Figure 2 shows, conserved introns are found within the N-terminal domain (HNL class; phase 0), between the N-terminal domain and the NBS domain (HNL, PNL and TNL classes; phase 0 for HNL and PNL, but phase 2 for TNL), and between the NBS domain and the LRR domain (HNL, PNL and TNL classes, all phase 0). These intron characteristics can be efficiently used to distinguish NBS-encoding genes from different classes. For example, a few divergent NBS-encoding genes in *P. patens* were difficult to classify at first by homology test and the phylogenetic method, but their intron characteristics later provided clear clues for their final classification within TNL class. The facts of these introns located between the TIR/PK/α/β-hydrolase, NBS and LRR domains are in line with the exon/domain shuffling theory of modular proteins. This theory advocates that ancient chimerical genes could have evolved by exon/domain shuffling through the transposable elements in their flanking introns. The formation of multi-domain proteins was the consequence of some more ancient single-domain proteins being brought together. This model seems to explain the origin of these three classes of NBS-encoding genes, although more direct evidence needs to be discovered.

Different from the other three classes, the CNL class is the only class of NBS-encoding genes showing no conserved domain-boundary introns, which is a key character to identifying a CNL class member. Considering such obvious differences, it is not surprising when the phylogenetic analysis (Figure 3B) found that the three intron-containing classes (HNL, PNL and TNL) showed a closer relationship and form a monophyletic super-class (with support value of 91), while the CNL class is sister to the super-class. The constructed phylogeny also suggests that the CNL class had originated at least in the common ancestor of land plants, as the liverwort *M. polymorpha* CNL genes and some of the moss *P. patens* CNL genes can form a strongly supported monophyletic group, reflecting at least one CNL gene present in their common ancestor and vertically inherited into both the liverwort and the moss lineages. The emergence of the other three classes of NBS-encoding genes seem to have coincided with the divergence of major lineages, and have resulted in the lineage-specificity observed for HNL in liverworts and PNL in mosses. The TNL class is more widespread, since they have been reported in mosses, gymnosperms and angiosperms. Nonetheless, the TNL class also shows sort of lineage specificity, as indicated by the yet unexplained absence of this class of genes in monocots [33].

Traditionally, NBS-encoding genes are divided into TNL and non-TNL classes, mainly based on the knowledge of angiosperm species. The CNL class is regarded as a major type of the non-TNL class. If this traditional way is followed, the newly discovered HNL and PNL classes in this study should be considered as two other non-TNL classes. However, both the constructed phylogeny and the intron analyses, as demonstrated above, have revealed a clear relationship among the HNL, PNL and TNL classes.

CNL class doesn’t have conserved introns, and has been found in all sequenced land plant species, including *M. polymorpha* we investigated in this study, suggesting an ancient origin as well as the necessity of its function by wide land plant groups. Differently, the two novel classes we found seem to present specifically in certain groups and reflected their functional restrictions. Further functional study on the genes of the two classes will help to explore their lineage-specificity.

## Materials and Methods

### Genome-wide Analysis of NBS-encoding Genes in *P. Patens*

*P. patens* assembly and gene models were obtained from the Joint Genome Institute data repository (ftp://ftp.jgi-psf.org/pub/JGI_data/phytozome/v7.0/Ppatens). To identify NBS-encoding genes in this moss genome, both BLAST and hidden Markov model searches were performed following the same procedures used before [13], [15], [34] First, possible homologs encoded in plant genomes were searched using BLASTp with the amino acid sequence of the NBS/NB-ARC domain (Pfam: PF00931) as a query. Second, the amino acid sequence of the first hit was checked and its NBS domain sequence was used as a modified query to conduct a second search. This second step was required because the standard amino acid sequence of the NBS/NB-ARC domain (Pfam: PF00931) was too divergent for searches in a bryophyte genome like *P. patens*, although it worked well within angiosperms. The expectation value threshold was set to 1.0, a value determined empirically to filter out most of the spurious hits. The Multiple Expectation Maximization for Motif Elicitation tool was used to analyze motif structures among NBS-encoding genes [35]. CC domains were detected using COILS with a threshold of 0.9 [36].

### NBS Fragment Amplification by PCR Using Degenerate Primers

Fresh *M. polymorpha* tissue samples were collected from field in Yangzhou, Jiangsu Province, China. Total cellular DNA was extracted by the CTAB method [37] and purified by phenol extraction to remove proteins. Genomic DNA fragments spanning conserved NBS sequences were amplified from the extracted DNA using a total of 25 oligonucleotide primers (Table S1). All primers were designed using *P. patens* NBS-encoding genes as references. Different primers pairs were designed to amplify different types of NBS-encoding genes. Most of the primers were designed to amplify 700 bp fragments from the P-loop to the RNBS-D motif covering ∼80% of the NBS domain. The Takara LA taq (Takara, Dalian, China) is a proof reading polymerase, and it was used for amplification to avoid PCR errors. The standard PCR methods were used: 3 min of DNA denaturation at 94°C, followed by 35 cycles of 30 s at 94°C, 30 s at 52°C, and 60 s at 70°C for each cycle. The last cycle was followed by 10 min of extension at 70°C. The amplified PCR products were further extracted using the gel extraction kit (Qiagen, Valencia, CA, USA) and cloned using the TOPO TA cloning kit (Invitrogen, Carlsbad, CA, USA). The selected clones were sequenced on an ABI3730XL using ABI Big-Dye technology (Applied Biosystems, Carlsbad, CA, USA). To guarantee saturated sequencing, 10–40 clones were picked for sequencing as needed for each cloning product. All unique genes were the products of three independent PCRs.

### Reverse Transcriptase–polymerase Chain Reaction and Rapid Amplification of 5′- & 3′-cDNA Ends

The first strand cDNA was synthesized from 5 µg of RNA with Superscript III reverse transcriptase (Invitrogen, Carlsbad, CA, USA) in a volume of 20 µl with oligo-dT primers. With the first strand cDNA as a template, the target gene fragments were amplified using gene specific RACE primers (Table S2), then cloned and sequenced as described above. The sequenced data from cDNAs were used to determine candidates for the specific amplification of their N- or C-terminal regions via 5′- or 3′-RACE. This method was used to obtain the full-length coding sequence of a gene. RNA was treated as per the protocol of the GeneRacer kit (Invitrogen, Carlsbad, CA, USA) and reverse transcribed into first strand cDNA. With the synthesized cDNA as a template, the 5′- or 3′-cDNA ends of the gene were amplified, cloned and sequenced.

### Sequence Alignment and Phylogenetic Analysis

Sequences were assembled and edited using Sequencher 4.2 (Gene Codes Corp., Ann Arbor, MI, USA), and were deposited in the NCBI under accession numbers JQ764686–JQ764728. Multiple alignments of amino acid sequences were performed by MUSCLE [38] with default options [39] and then by MEGA 5.0 for manual corrections of the alignments [40]. The resulting amino acid sequence alignments were used to guide the alignments of the nucleotide coding sequences. Phylogenetic analysis was conducted using maximum likelihood method with the RAxML 7.0.4 program. The GTR+I+G model was used to establish the best tree and a total of 100 rapid bootstrap replicates were performed [41].

## Supporting Information

Table S1 PCR primers for the amplification of the NBS-encoding genes in *Marchantia polymorpha*.
(DOC)

Table S2 5′- and 3′-RACE Primers for the NBS-encoding genes in *Marchantia polymorpha*.
(DOC)
