# Supplementary material for: A Primary Survey on Bryophyte Species Reveals Two Novel Classes of Nucleotide-Binding Site (NBS) Genes
Source: PLoS One. 2012 May 15;7(5):e36700. doi: 10.1371/journal.pone.0036700 (PMC3352924; doi:10.1371/journal.pone.0036700)
Supplement: Table S1 — PCR primers for the amplification of the NBS-encoding genes in Marchantia polymorpha. (DOC) [file pone.0036700.s001.doc]

Table S1: PCR primers for the amplification of the NBS-encoding genes in *Marchantia polymorpha*.

| Class | Primer name | Oligonucleotide primer sequence (5′–3′) |
| --- | --- | --- |
| CNL | Mar-CNL-F0a | GGGAGAGGAGGAGCMGGTAARACC |
|  | Mar-CNL-F0b | GGAGGAGAYGGGAAYGTGGAAGAWGCAAAGVA |
|  | Mar-CNL-F1 | GCCTCAAGACTGTYAAATGATCYCCMA |
|  | Mar-CNL-F2 | CTGGTGTTGGATGATGTATGGAAGTC |
|  | Mar-CNL-R0a | GTCTTCGGGAWAYGCWGCAAAGTAGTAGAA |
|  | Mar-CNL-R0b | GTCTTCGGGATACGCAGCAAAGTA |
|  | Mar-CNL-R1 | GTAGTAGAAGCAATCTTTCAAATGACGA |
|  | Mar-CNL-R2 | AACCCAGTGTTGACGATTTRTCCGAAA |
|  | Mar-CNL-R3 | TCCRATGACTTTCAGGSCTAAGGGRAGC |
| nonCNL | Mar-F0 | TTTGSGGSATGGKWGGARTWGGGAAAAC |
|  | Mar-R0 | GCRRCRTCAAGGAAYATWTYYTTCTC |
|  | Mar-F1 | ATGGKWGGARTWGGGAAAACHACWCTC |
|  | Mar-F2a | GGCATGGGTGGAATTGGNAARACNAC |
|  | Mar-F2b | GGGATGGTAGGAGTTGGNAARACNAC |
|  | Mar-F2c | GCATCAGGCGGGGCCGGNAARACNYT |
|  | Mar-F3a | TTTGCGGCATGGGDGGAATA |
|  | Mar-F3b | TTGGGATTTTGGGGGATGGT |
|  | Mar-F4a | GGTAAAACTACAYTNYTNAA |
|  | Mar-F4b | ACTACACTGCTTAAYGARATHGT |
|  | Mar-R1 | GAADGMDGCARGATCAAGGAAYATWT |
|  | Mar-R2a | GABGCARCATCAAGGAACAT |
|  | Mar-R2b | ACCTCTAGGGTTAANGGNAGNCC |
|  | Mar-R2c | ACTTTCAGCGCCARNGGNAGNCC |
|  | Mar-R3a | GGCAGACCCTCACAGAGNGGNARRAA |
|  | Mar-R3b | AAAGGTTGGTTGTCTGGGAA |
