# Supplementary material for: A Primary Survey on Bryophyte Species Reveals Two Novel Classes of Nucleotide-Binding Site (NBS) Genes
Source: PLoS One. 2012 May 15;7(5):e36700. doi: 10.1371/journal.pone.0036700 (PMC3352924; doi:10.1371/journal.pone.0036700)
Supplement: Table S2 — 5′- and 3′-RACE Primers for the NBS-encoding genes in Marchantia polymorpha. (DOC) [file pone.0036700.s002.doc]

Table S2: 5’- and 3’-RACE Primers for the NBS-encoding genes in *Marchantia polymorpha*.

| Class | Primer name | Oligonucleotide primer sequence (5’–3’) |
| --- | --- | --- |
| HNL | 3’-HNL-F1 | TTTGSGGSATGGKWGGARTWGGGAAAAC |
|  | 5’-HNL-B-2-R1 | CGCCCACAACTTCTCATCCAGACTCACT |
|  | 5’-HNL-B-2-R2 | CGACGATCTTGTGTACCCATTCCTTCAG |
|  | 5’-HNL-D-2-R1 | GGTGCTGGGGTAATCTTCTTGCCTTTG |
|  | 5’-HNL-D-2-R2 | CACCAGGAATCTGCTTGACGCCCTCA |
| CNL | 3'-CNL-2-F1 | CCCCCACCCCCCAGTTCCGAACAGGACTA |
|  | 3'-CNL-2-F2 | GCTGGTGTTGGATGATGTATGGGAGTGGAGAA |
|  | 5’-CNL-R1 | CCCATACATCATCCAACACCAGCAACAG |
|  | 5‘-CNL-R2 | CTGGGGGGTGGGGGCAGCATTCTCAACA |
|  | 5’-CNL-R3 | GCTCCCAGACGAAGATGGATGATTCA |
